# Supplementary material for: Changes of Viscoelastic Properties of Aptamer-Based Sensing Layers Following Interaction with Listeria innocua
Source: Sensors (Basel). 2021 Aug 19;21(16):5585. doi: 10.3390/s21165585 (PMC8402281; doi:10.3390/s21165585)
Supplement: Supplementary file 1 [file sensors-21-05585-s001.zip › sensors-1282375-supplementary.pdf]

Changes of Viscoelastic Properties of Aptamer-Based Sensing Layers Following Interaction with *Listeria innocua*

Marek Tatarko[1], Sandro Spagnolo[1], Veronika Oravczová[1], Judit Süle[2], Milan Hun[2], Attila Hucker[2], Tibor Hianik[1*]

[1]Department of Nuclear Physics and Biophysics, Faculty of Mathematics, Physics and Informatics, Comenius University in Bratislava, Mlynská dolina F1, 842 48 Bratislava, Slovakia; tatarko4@uniba.sk (M.T.); spagnolo2@uniba.sk (S.S.); oravczova2@uniba.sk (V.O.)
[2]Hungarian Dairy Research Institute Ltd., 9200 Mosonmagyaróvár, Hungary; jsule@mtki.hu (J.S.); mhun@mtki.hu (M.H.); ahucker@mtki.hu (A.H.)
*Corresponding author: tibor.hianik@fmph.uniba.sk

Supplementary Material

1. Changes of the frequency and dissipation following non-specific interactions of *E. coli* with the neutravidin layer

In order to check possible interaction of bacteria with only neutravidin monolayer, we measured changes in frequency and dissipation following the addition of various concentrations of *E. coli*. The results are presented on the Figure S1.

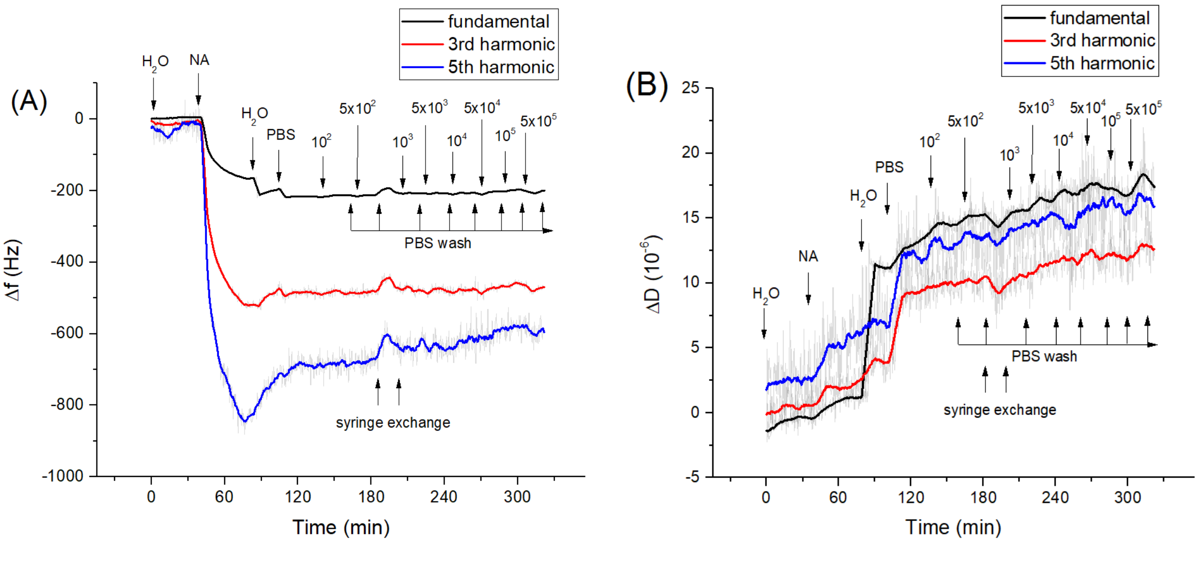

Figure S1. The kinetics of the changes of (A) fundamental frequency, 3rd and 5th harmonic frequencies, respectively) and (B) dissipation vs. time following addition of neutravidin (NA) and *E. coli* (in CFU/mL). The moment of addition of NA and bacteria and washing of the sensing surface by deionised water, PBS as well as exchange of syringe in the syringe pump are shown by arrows.

2. Changes of the frequency and dissipation following addition of *Listeria innocua* to the sensing layer

Figure S2 shows representative plot of the kinetics of the changes of resonant frequency, Δf, and dissipation, ΔD, following addition of *Listeria innocua* to the surface of sensing layer composed of

biotinylated DNA aptamers connected to the neutravidin monolayer chemisorbed at QCM transducer. At the frequencies higher that $5x10^5$ CFU/mL the increase of resonant frequency and decrease in dissipation has been observed. This can be explained by the behavior of bacterial layers as a coupled oscillator system (See Figure S3 for explanation).

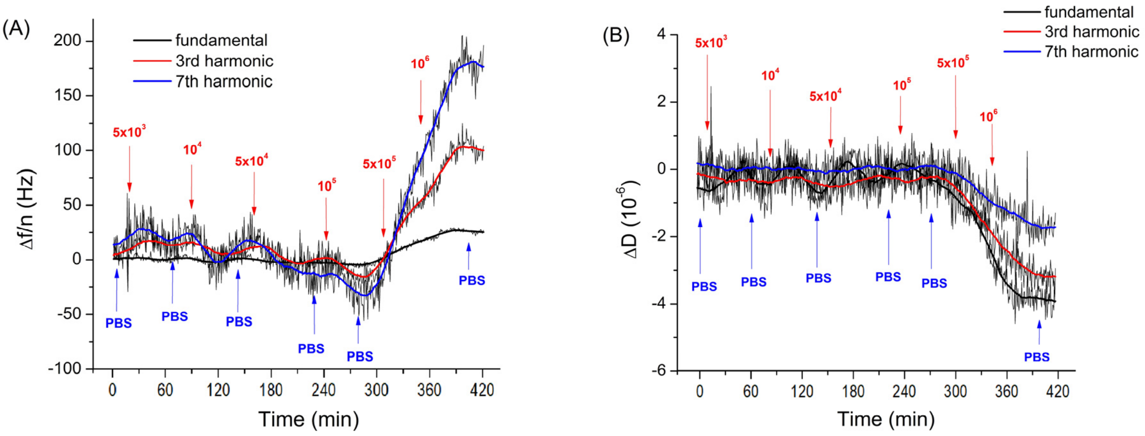

Figure S2. Representative kinetics of the changes of (A) fundamental frequency, 3rd and 7th harmonic frequencies (values divided by their harmonic number n = 3, 7, respectively) and (B) dissipation vs. time following addition of *Listeria innocua* (in CFU/mL). The moment of addition of bacteria and washing of the sensing surface by PBS are shown by arrows.

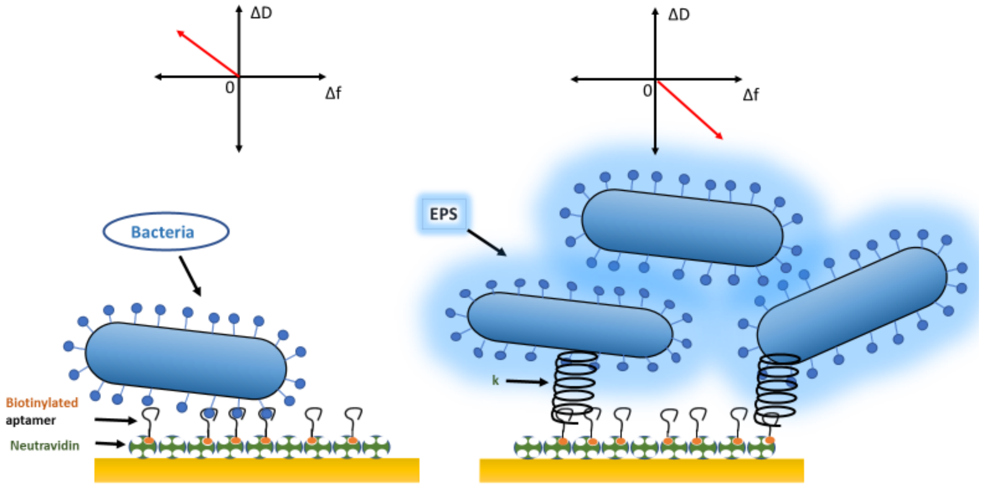

Figure S3. The scheme of the specific interaction of bacteria *Listeria innocua* with the sensing layer composed of biotinylated DNA aptamers immobilized at the neutravidin monolayer chemisorbed at QCM transducer (left). The positive shift of the resonant frequency (right) can be explained by coupled oscillator theory. Depending on the stiffness, k, of the spring connected the sensing layer with bacteria, the frequency shift can be more or less positive. The released extracellular polymeric substance (EPS) can also affect the frequency shift. However, it is closely connected with the QCM substrate and may cause increased mass loading and thus the negative frequency shift. Upper panel schematically illustrate the direction of the changes of resonant frequency, Δf, and dissipation, ΔD.
